# C-H Groups as Donors in Hydrogen Bonds: A Historical Overview and Occurrence in Proteins and Nucleic Acids

**DOI:** 10.3390/ijms241713165

**Published:** 2023-08-24

**Authors:** Zygmunt Stanislaw Derewenda

**Affiliations:** Department of Molecular Physiology and Biological Physics, School of Medicine, University of Virginia, Charlottesville, VA 22903-2628, USA; zsd4n@virginia.edu; Tel.: +1-434-243-6842

**Keywords:** hydrogen bonds, C-H…O bonds, protein structure, nucleic acid structure, science history

## Abstract

Hydrogen bonds constitute a unique type of non-covalent interaction, with a critical role in biology. Until fairly recently, the canonical view held that these bonds occur between electronegative atoms, typically O and N, and that they are mostly electrostatic in nature. However, it is now understood that polarized C-H groups may also act as hydrogen bond donors in many systems, including biological macromolecules. First recognized from physical chemistry studies, C-H…X bonds were visualized with X-ray crystallography sixty years ago, although their true significance has only been recognized in the last few decades. This review traces the origins of the field and describes the occurrence and significance of the most important C-H…O bonds in proteins and nucleic acids.

## 1. Introduction

Hydrogen bonds (H-bonds) constitute one of the most significant and consequential non-covalent interatomic interactions in biological molecules. They are responsible for the stabilization of the main secondary structure elements in proteins and for the complementarity of the two strands in DNA and RNA. H-bonds play important roles in the interactions between enzymes and their substrates/products, in the recognition of antigens by antibodies, and in many other biological phenomena. Although initially, the H-bond was thought to occur only between electronegative atoms, i.e., mainly O and N, decades of research uncovered a range of often weaker, yet very important interactions, in which polarized C-H groups are donors. Of these, the C-H…O bond is the most ubiquitous and significant. First proposed in 1936 to occur between acetone and chloroform, this type of an H-bond was controversial for several decades, until contemporary advanced spectroscopic and crystallographic methods provided compelling data supporting its chemical nature and significance. Moreover, these interactions were found to be ubiquitous in biological compounds, including proteins and nucleic acids, with important functional consequences. The purpose of this review is to provide an overview of the history of the field, including lesser-known pioneering publications, and to discuss the experimental and theoretical support for the presence and function of the C-H…O bonds in nucleic acids and proteins.

## 2. History

### 2.1. Early Period: Emergence of Concepts

The concept of a hydrogen bond (H-bond)—and specifically the O-H…O bond between water molecules—was first proposed in 1920 by Wendell Latimer and Worth Rodebush, two young scientists working at the College of Chemistry directed at Berkeley by Gilbert N. Lewis [1,2]. It was, in fact, Lewis who coined the term ‘*hydrogen bond*’ three years later [3,4]. An analogous hypothesis, promoting an intramolecular bond of the same nature, was proposed independently by Maurice L. Huggins, also at Berkeley [5,6]. With the advent of the concept of electronegativity, introduced by Linus Pauling in 1932 [7], the H-bond was initially seen primarily as an electrostatic phenomenon, and C-H groups were not considered to be involved. A hypothesis departing from this view was introduced by an English physical chemist, Samuel Glasstone, at the Faraday Society meeting in Edinburgh on 24–26 September 1936.

Samuel Glasstone (1897–1985) received a PhD (1922) and DSc (1926) in chemistry from the University of London and, in 1929, became a lecturer in chemistry at the University of Sheffield. His interests focused on the properties of the mixtures of polyhalide organic compounds, e.g., polyhalomethane—and specifically chloroform—with oxygen-containing organic compounds such as ether and acetone. Interestingly, there is a long history of studies in this field. Chloroform was synthesized in 1831, and by 1834, the French chemist Jean-Baptiste Dumas showed that it contains hydrogen, determined the formula, and gave the compound its name. James Young Simpson used the compound in 1847 for the first narcosis—on himself. Chloroform is poorly soluble in water but highly soluble in ethanol, ethyl ether, and acetone. The so-called ACE mixture (alcohol, chloroform, and ether, typically in a 1:2:3 ratio) has been used for anesthesia since around 1860. It was soon discovered that the mixtures of halogenated carbohydrates with ether and ketones showed large deviations from Raoult’s Law [8,9], which defined an ideal solution as one in which interactions between all molecules are of the same nature. Such deviations were rationalized—somewhat speculatively—by a tendency of the components, e.g., chloroform and acetone, to interact, forming a stable complex [10,11,12]. This was hypothesized by the Hungarian physical chemist Friedrich Dolezalek, even prior to the publication of the theory of the chemical bond by Lewis [13]. Importantly, it was discovered that trihalomethanes form complexes with ether or acetone more readily than tetrahalomethanes, implicating hydrogen in the formation of the putative complex. In 1929, Wilfred F. Wyatt, working at the University of Sheffield and a colleague of Glasstone, discovered that acetone and chloroform form a crystalline compound (CH_3_)CO·2CHCl_3_ [14]. Interestingly, Wyatt attributed the formation of such complexes to the possibility of the oxygen donating a free electron pair to the halide—not the hydrogen. In 1936, Emyr A. Moelwyn-Hughes and Albert Sherman (University of Cambridge) published a paper in which they discussed a classification of chemical bonds [15]. Although the H-bond is conspicuously missing from the list, the acetone–chloroform interaction is explicitly defined as a head-on dipole interaction involving a polarized C-H group of chloroform [15].

It was shortly thereafter, in September 1936, that Samuel Glasstone made the explicit suggestion in his talk in Edinburgh that the chloroform’s C-H group acts as a donor in an intermolecular H-bond with acetone’s oxygen. The meeting of the Faraday Society was dedicated to the subject of ‘*Structure and Molecular Forces in (a) pure liquids and (b) solutions*’. In his presentation, Glasstone suggested that the C-H bond in chloroform is polarized, although he maintained that this is because oxygen in C=O repels the electron pair ion C-H. The meeting was well attended, and a vibrant discussion followed each presentation, which survived in redacted form. Several of the attendees, including John D. Bernal—who was already a renowned expert in the field of H-bonds [16]—were critical of the suggestion of a C-H…O bond and argued that the interaction is purely electrostatic. This is very likely the reason why Glasstone’s published paper, which came out nearly a year after the meeting [17], was careful not to use the term ‘hydrogen bond’ even though the description leaves no doubt about what he thought.

The Faraday Society meeting was attended by twenty-six overseas participants, although the list of names was not published. One of them was very likely Maurice L. Huggins, the coauthor of the concept of the classical hydrogen bond [1,2,4]. One month after the meeting—and so nearly a year before the talks and summary of discussion came out in the press—Huggins submitted a broad review of what he called ‘hydrogen bridges’, which included a section on C-H…O interactions, explicitly quoting Glasstone’s talk [18].

In a parallel development, in December of 1936, Glenn F. Zellhoefer, a scientist working for the Williams Oil-O-Matic Heating corporation in the USA, submitted a paper to a rather obscure journal, with voluminous data on the solubility of halogenated hydrocarbon refrigerants in organic solvents [19]. His purpose was to find the best combination for the use in refrigeration industry, and he did not pursue any explanation for the observed deviations from Raoul’s Law. However, Zellhoefer then entered into a collaboration with a group of physical chemists from the Chemistry Department of the University of Illinois (and subsequently the renowned Noyes Chemical Laboratory), namely Michael J. Copley and Carl S. Marvel. The latter was elected in 1938 to the National Academy of Sciences and was a winner of numerous awards for his broad-ranging research of polymers [20]. The three published together four papers in 1938, starting a series titled ‘*Hydrogen bonds involving the C-H link*’. The first paper in the series was the first use in the literature of the term ‘*hydrogen bond*’ applied to an interaction where the C-H group is a donor [21]. This paper, and three that followed it [22,23,24], extended Zellhoefer’s original experimental data and rationalized it in the context of the C-H…O bonds, explicitly citing Wyatt, Glasstone, and Huggins. They showed evidence supporting C-H-mediated bonds involving, among others, dichloromethane and methylene chloride. Ten more papers followed, for a fourteen-paper series [25,26,27,28,29,30,31,32,33,34]. Unfortunately, because a number of these papers are not listed in citation databases, the contribution from the Zellhoefer–Copley team has not been fully acknowledged in the literature. Together, they constitute a compelling body of work providing extensive evidence of polarized C-H group serving as donors of H-bonds to a range of acceptors. The last paper in the series, from 1941, was authored by Carl S. Marvel and J. Harkema (the only one not coauthored by Michael J. Copley, and one of the two not coauthored by Zellhoefer) and showed that the C-H group of triphenylmethane was not a donor [34].

This body of work was known—despite the ongoing World War II—in the UK. The first major review of hydrogen bonding published after the war, by Louis Hunter, a professor and founder of the Chemistry Department at the University of Leicester [35], provided a comprehensive bibliography supporting the notion of C-H as donors in H-bonds and citing contributions from the Noyes Chemical Laboratory.

### 2.2. Spectroscopic Evidence

As we have seen, the hypothesis of the existence of C-H…O(N) bonds was initially inferred in a rather speculative way from the macroscopic properties of solutions, such as solubility or boiling points. However, two experimental breakthroughs soon provided badly needed direct evidence using spectroscopy and targeting the bonds themselves. In 1938 and 1939, Walter Gordy (1909–1985), a pioneering expert in microwave spectroscopy and a long-time professor at Duke University, published three papers providing the first spectroscopic infrared evidence of a C-H…O bond between chloroform and acetone or dioxane [36,37,38]. This study was extended by George C. Pimentel (1922–1989), a professor at the University of California, Berkeley, who observed infrared evidence of the H-bond between chloroform and carbonyl of amides [39]. Moreover, he estimated the energy of this interaction to be ~2 kcal/mol. Pimentel—who went on to discover a chemical laser—was also the first to use NMR in 1955 to obtain corroborating data on the interaction of chloroform with acetone and triethylamine [40].

In 1960, the first textbook on hydrogen bonding was published by Pimentel and McClellan [41], with a comprehensive bibliography. The chapter on C-H groups concluded that: ‘*(…) the evidence in favor of association of chloroform with bases is conclusive. The evidence that this association is of the H-bonding type is substantial, and is consistent with the statement that chloroform forms H-bonds, at least with such strong bases as pyridine and trimethylamine*’. The authors were more doubtful about other systems. The same year, Campbell and Kartzmark determined the free energy of the chloroform–acetone H-bond to be 2.7 ± 0.1 kcal/mol, close to Pimentel’s original estimate [42].

In June 1963, Adam Allerhand and Paul von Rague Schleyer of Princeton University used infrared spectroscopy to study a range of compounds to establish which C-H groups have the ability to serve as donors [43]. They concluded that the H-bonding propensity is highest for the *sp* carbon, followed by *sp^2^* and *sp^3^*. They emphasized the role of electronegative groups adjacent to carbon in the polarization of the C-H bond, but they also made the following point: ‘*It is apparent from the present survey that C-H groups can have a very wide range of proton donor abilities. C-H spectral shifts range from 0 to well over 100 cm^−1^ and in some cases are comparable even in absolute magnitude to OH spectral shifts involving methanol as proton donor*’. This prescient article remains one of the most cited papers today in the field of C-H…X H-bonds from the early period.

### 2.3. The Turning Point: Structural Evidence from Crystallography

While spectroscopic studies were of paramount importance in that they clearly showed electronic changes in specific C-H…X interactions in solution, such interactions were yet to be visualized. X-ray crystallography was still in an early stage, and even though the number of organic structures characterized by X-ray diffraction grew quickly, the relatively low precision of these studies did not routinely allow for the confident positioning of hydrogen atoms, although progress was being made. By 1951, Dulmage and Lipscomb published the crystal structure of hydrogen cyanide [44] and found it to be arranged as expected, end-to-end with the C…N distance of 3.18 Å, and the estimated N…H distance (hydrogen was not directly detected in their study) was 2.1 Å, well below the sum of van der Waals radii. Short C-H…X distances were found in other structures, but no general conclusions were drawn until 1962. That year, D. June Sutor (1929–1990), a crystallographer from New Zealand working at Birkbeck College in London with John D. Bernal, published a seminal short paper in *Nature* in which she cataloged short intra- and intermolecular C…O(N) distances identified in eight crystal structures, including that of 1,3,7,9-tetramethyluric acid, which Sutor herself had just solved [45,46]. In those few cases where the positions of the hydrogen atoms were explicitly determined (calculations involving ‘riding’ hydrogens were introduced much later), she characterized the stereochemistry of the C-H…O(N) interactions [46]. The distances between carbon and oxygen (or nitrogen) were shorter than the sum of the van der Waals radii (assumed to be 3.3 Å). Similarly, the distances between H and O were in some cases significantly shorter than the sum of atomic radii, assumed by Sutor to be 2.6 Å (the correct value is 2.72 Å). This stereochemistry strongly suggested the formation of H-bonds. An expanded version of the study including 24 structures was published by Sutor in 1963, with the same overall conclusion [47]. She specifically pointed out the higher propensity of the methine groups (i.e., =CH-) within aromatic systems to form H-bonds, including one in the crystal structure of cytidine (see below) [48]. With remarkable prescience, Sutor posits that C-H…O bonds may be significant in biology [47]. Interestingly, she did not expand in her papers on the corroborating evidence from spectroscopy and physical chemistry; she simply references the textbook by Pimentel and McClellan, and the references therein, so she was certainly aware of the previous work.

Citation records show that Sutor’s papers were well received and immediately cited by many authors. Importantly, several high-impact papers focusing on the structure of nucleotides and nucleosides reported observations consistent with Sutor’s conclusions [49,50,51,52]. In the meantime, Sutor left the field of H-bonds, went briefly back to New Zealand, and then returned to England in 1966 to work with Dame Kathleen Lonsdale on kidney stones, an area in which she continued to research until the early 1980s.

Five years after Sutor’s papers, in 1968, Jerry Donohue (1920–1985)—an American chemist who earned his PhD under Linus Pauling and moved to Cambridge in 1952 to share an office with Francis Crick and James Watson—published a chapter in a book edited by Rich and Davidson commemorating Pauling’s work, with a section titled ‘*The C-H…O Hydrogen Bond: What is it?*’. Donohue was regarded as an authority on H-bonds, having published, among others, a highly regarded review of the field with respect to organic compounds [53]. Moreover, it was Donohue who pointed out to James Watson the correct tautomeric forms of DNA bases, thus enabling Watson to build the DNA double-helix model (see below). Donohue was very conservative in his interpretation of H-bonds and argued that proximity need not be taken as evidence of cohesive interactions. In the 1963 book chapter, he cited the famous Ramachandran’s paper [54], which—among others—stated that, in polypeptides, H…O distances can be as short as 2.2 Å in the absence of an H-bond (which is correct). He then selected and cited one specific sentence from Louis Hunter’s 1946 review, which stated that ‘*C-H…O putative bonds are so weak that they exist only in the context of activating groups*’. While the citation is correct, Hunter made an overstatement which is in conflict with other fragments of the review, and there was sufficient evidence published (including the paper by Allerhan and von Rague Schleyer [43]) demonstrating that these bonds exist in a range of systems. Donohue also questioned the precision of several structures used by Sutor, which indeed was deficient, and concluded simply that C-H…O bonds do not exist. Like Sutor, Donohue did not discuss evidence from spectroscopy or physical chemistry—to him, it was primarily a stereochemical argument.

It has been argued that Donohue’s 1968 chapter was a misogynistic attack on Sutor and that it stalled the field of C-H…X bonding [55,56]. While it is true that Donohue enjoyed a strong reputation and that his article received some laudatory reviews, there is no evidence supporting this view. Sutor’s two papers continued to attract interest and were widely cited. Nor did the field stall. Soon after Donohue’s chapter was published, Pedro Olympia from Tufts published a theoretical study of C-H distances and stretching frequencies of the relevant H-bonds [57]. In 1974, a book was published by R.D. Green (University of Saskatchewan) that was dedicated entirely to hydrogen bonding by C-H groups [58]. The book presents a comprehensive, encyclopedic overview of all studies conducted in the field; its last chapter, titled ‘*The Nature of C-H Hydrogen Bonding*’, expands primarily on the spectroscopic and NMR characterization of C-H…X bonds. Its bibliography includes 409 items.

Experimental support continued to accumulate, including a novel approach to quantum chemical calculations [59,60,61]. Crystallographic evidence was comprehensively revisited in 1982 by Robin Taylor and Olga Kennard [62]. Their paper presented a detailed stereochemical analysis of 113 crystal structures obtained via neutron diffraction, a database that overcame all the purported weaknesses of Sutor’s analysis. The majority of the structures clearly suggest the presence of cohesive C-H…O bonds, with the proton within 30° of the plane of *sp^2^* oxygen orbitals, when carbonyl oxygens are involved. The authors pointed out that there is a propensity of H-bonds in which the donor C-H group is adjacent to nitrogen. Examples of N and Cl as acceptors were also identified. This pivotal paper has been cited to date over 2500 times (according to the https://www.webofscience.com database) and constitutes a milestone in the evolution of the field of H-bonds.

### 2.4. Recent Advances

Following the study by Taylor and Kennard, another group to focus on the subject of C-H…O bonds was that of Gautam Desiraju, who was then at the University of Hyderabad [63,64]. He became particularly interested in the occurrence of such bonds in intermolecular contacts in crystals and the possibilities of crystal engineering [65,66]. In 1991, Desiraju published an article under the same title as the controversial chapter penned by Donohue more than two decades earlier: ‘*The C-H…O Hydrogen Bond in Crystals: What is it?*’. In contrast to Donohue, Desiraju provides a comprehensive, multidisciplinary discussion of the evidence and rigorous assessment of crystallographic data, which by then grew exponentially, and its accuracy was dramatically increased [56]. His answer to the title question was ‘*It certainly is*’. By 1999, Gautam Desiraju and Thomas Steiner published the second book in the field: ‘*The Weak Hydrogen Bond in Structural Chemistry and Biology*’.

The field then took one more unexpected turn. For a long time, it was understood that H-bonding results in the redshift of D-H stretch vibration (where D is a donor atom), consistent with the lengthening and weakening of the bond. In fact, it was noted that the magnitude of the redshift and the strength of the H-bond are correlated. However, in 1989, a study was published that described an accidentally recorded blueshift in the C-H group of chloroform in triformylmethane [67]. This result went unnoticed until 1997, when another group measured similar spectra, and this time observed a pattern in the stretch vibrations in C-H/D in a range of haloforms [68]. Further studies showed that other donor groups possess similar properties and provided an explanation [69,70,71]. In simple terms, the electron affinity of the donor atom (Y) bound to hydrogen may cause an increase in electron density in the presence of an acceptor and cause the Y-H bond to contract. This is normally balanced and superseded by the attraction between H and the electronegative acceptor. The final balance depends on the chemical context of the donor–acceptor system, although in a vast majority of cases, i.e., canonical bonds and some weak C-H-mediated bonds, it is strongly biased toward a redshift [72].

The C-H…X hydrogen bond was finally ‘legitimized’ by the new general H-bond definition introduced by IUPAC in 2011 [73]. The broad definition left no doubt that chemistry recognized a spectrum of interaction that together can be defined as a hydrogen bond, irrespective of its strength.

Chloroform continues to serve as a model system for C-H donor groups. Vibration inelastic neutron scattering spectra confirmed that chloroform–acetone exists as an independent moiety bonded via C-H…O contact [74]. While longer and weaker than O-H…O bonds, C-H…O is able to play a key role in supramolecular structures. More recently, linear and non-linear infrared spectroscopy revealed the nature of the H-bond between chloroform and the carbonyl oxygen of an amide [75]. The 2DIR investigations of the chloroform/amide solution revealed the presence of chemical exchange cross-peaks between two different hydrogen-bonded states of the amine. This work demonstrates how chloroform associates with the carbonyl oxygen of the amide in agreement with the directionality of a hydrogen bond [75].

There is intense interest in the characterization of even weaker interactions, those involving *sp^3^* C-H groups, which until fairly recently were discounted as completely insignificant. A recent high-impact publication in *Science* demonstrated charge transfer across the C-H…O bonds between water and alkyl hydrogens at the water–hexadecane interface, using sum–frequency scattering spectroscopy [76]. Another study, using temperature-dependent Raman spectroscopy with quantum chemical calculations, targeted the blue-shifted CH_3_ stretching vibration in a methanol–water mixture [77]. The authors also found that the *sp^3^* C-H…O interactions are enhanced with increased temperature, in contrast to conventional H-bonds. Finally, vibration solvatochromism was introduced to investigate the C-H…O bonds in liquid solutions [78]. Studying a range of alcohols, the authors found an abnormal Raman blueshift in C-H and C-D bonds of alcohols in water, for both Cα and Cβ. This work strongly hints at the ubiquity of H-bonds between water and non-polarized C-H groups, with potential consequences for the protein-solvation shell structure [79]. New avenues of research are being pursued, including C-H…H-C interactions [80]. We apologize to many authors of relevant recent papers for not citing them due to space constraints.

As the knowledge and understanding of the nature of C-H…O H-bonds evolved, they became recognized as a major type of non-covalent interaction in biological molecules, including proteins and nucleic acids.

## 3. Significance of C-H…O H-Bonds in Biological Macromolecules

### 3.1. Earliest Hypotheses and Observations

The solution of the 3D atomic structures of nucleic acids (DNA and RNA) and proteins constitutes one of the most important scientific legacies of the 20th century. The structure of B-DNA was first proposed on the basis of stereochemical arguments and limited experimental data from fiber diffraction [81,82,83]. What is not often realized is that its full validation using single-crystal diffraction data had to wait another 27 years [84,85]. The research into structure and function relationships in various forms of DNA and RNA continues to date. Proteins were the subject of X-ray crystallographic investigations since it was discovered that—in spite of their size—they can crystallize and yield diffraction patterns, attesting to a high order of atomic structure [86,87]. It took 25 years to unravel the first structure, that of myoglobin [88]. The complexity of the structures of biological macromolecules precluded the application of simple spectroscopic or physical assays, which could yield evidence supporting the presence of C-H…X bonds in small molecules. Moreover, early crystallographic structures were typically solved using data at a medium resolution, and computing methods used to refine such structures were not developed until the late 1970s.

Interestingly, the first suggestion of the possible involvement of C-H…O bonds in protein structures predates any of the early crystallographic efforts and model building by Pauling and his associates [89,90,91,92]. In October of 1942, Maurice Huggins submitted one of the most prescient papers in the field of protein structure, contemplating the possible configuration of the polypeptide chain in fibrous proteins [93]. In it, Huggins discussed spectroscopic evidence supporting the existence of canonical hydrogen bonds between amide and carbonyl groups and then wrote:

*‘The hydrogen atoms of the CHR (i.e., CαHR—ZSD) groups may also form bridges to carbonyl oxygen atoms, since there is good evidence for C-H…O bridges in comparable structures (multiple references to the Copley/Zellhoefer series: ZSD). The possibility will be discussed in connection with the structure of collagen (Huggins later in the paper proposes such a structure—ZSD).’*

(Figure 1)

Although Huggins was correct on both counts, his paper was completely ignored; the H-bonds in question were discovered half a century later (see below).

**Figure 2:**
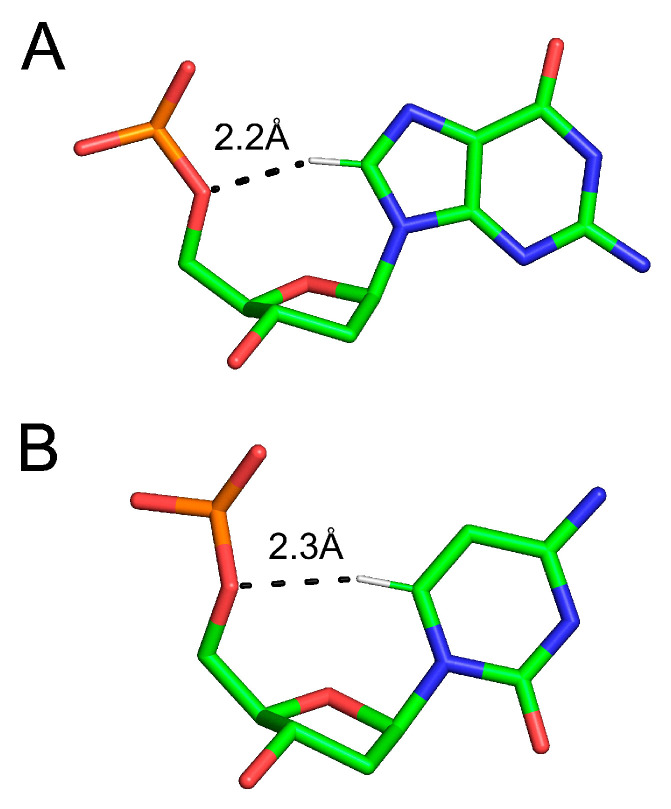
The deoxyribose-base H-bonds in the B-form DNA: (**A**) between the C(8)-H and O5′ in the adenosine nucleotide; (**B**) between C(6)-H and O5′ in the cytosine nucleotide first observed by Furberg in 1950. Colored according to atom type: green—carbon; blue—nitrogen; red—oxygen. Only the relevant hydrogen atom (white) is shown. The same color scheme is used in the remaining figures. The atomic models shown are extracted from the 1.2 Å resolution structure of B-form DNA [95].

The first hint regarding the presence of C-H…O bonds in nucleic acids came from the observation of the C(6)-H…O5′ interaction in cytidine, the crystal structure of which was solved in 1950 by Sven V. Furberg (1920–1985), three years prior to the proposal of double-helix structures [48] (Figure 2). He wrote:

*‘The distance between the atoms C4 (NB: the convention was later modified so that this is C6 according to accepted numbering—ZSD) in the pyrimidine and O5 ‘in the D-ribose is only 3.24 Å which is considered by the author to be significantly less than the normal van der Waals approach of 3.4–3.5 Å. This would seem to indicate some kind of attraction, possibly of the hydrogen bond type.’ (…) The reason for the formation of the bond may be sought in a possible polarization of the group (CH)_4_ by the electronegative substituents in the pyrimidine ring.’*

In the second paper Furberg published the same year [94], in which he used the structure of cytidine and several other results to speculate in general about the structure of nucleosides, he clarified that the polarization of the methine group in cytosine is due to the proximity of the keto and amino groups. He then made a point about the proposed structure of adenosine that no H-bond is there between C(8)-H and O5′, because ‘*the only substituent in the purine ring is far away from the group concerned*’ [94]. Thus, he made a serious error of judgment, discounting the presence of the two nitrogen atoms flanking C(8) in purines.

It is interesting to note that Furberg explicitly introduced the possibility of a C-H…O H-bond in cytidine and provided rationale (the polarization of the C-H bond), although he did not cite any relevant previous papers supporting this notion. His idea was ignored by Watson and Crick in their detailed description of the double helix [96].

The unique H-bond described by Furberg in cytidine was included among the cohort described by Sutor [46,47], and its presence was later reaffirmed in crystallographic studies of other purine and pyrimidine nucleosides and nucleotides, including adenosine-2′5′-uridine phosphate [97,98]. It was soon realized that the C(6)-H…O5′ and C(8)-H…O5′ bonds in pyrimidines and purines, respectively, constitute a ubiquitous feature stabilizing the *gauche, gauche* conformation of the sugar ring [99]. This was also visualized with the first X-ray structure of the yeast Phe tRNA at 2.5 Å resolution [100].

What follows is an overview of the most functionally important C-H…O bonds discovered so far in proteins and nucleic acids.

### 3.2. C-H…O Bonds in Proteins

Proteins offer a limited spectrum of chemistry with 20 amino acids, and there are only two heterocyclic ones, i.e., tryptophan and histidine, with significantly polarized methine groups. However, it is the Cα-H group—also polarized because of the proximity of the electron-withdrawing carbonyl and amide groups—that plays an important structural role in protein secondary structure. Other examples of C-H-mediated bonds, including aromatic C-H groups [101] and *sp^3^* carbon groups [102], have also emerged in recent years.

#### 3.2.1. Cα-H Groups as Ubiquitous Donors in H-Bonds in Proteins

**Figure 3:**
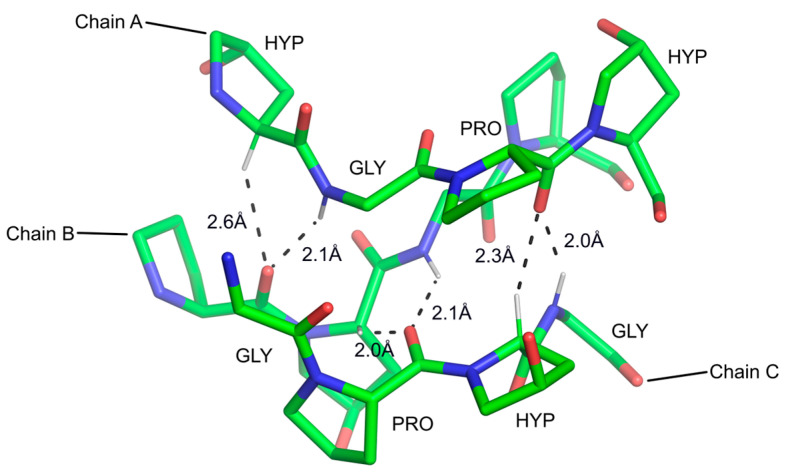
The two types of hydrogen bonds stabilizing the three chains (A, B, and C) in the collagen structure [109]. Both canonical and Cα-H…O=C bonds are shown with distances in Å. Only the residues in Chains A and C are labeled.

In 1993, Steiner and Saenger published a study of 46 neutron diffraction crystals containing water molecules [103]. They showed evidence from interatomic distances and angles that 8% of water molecules in crystals accept H-bonds from C-H groups, with H…O distances < 2.5 Å and 38% with distances < 2.8 Å (the sum of the van der Waals radii being 2.72 Å). Almost out of context, they showed a water cluster in the 1.8 Å X-ray crystal structure of the protein actinidin [104], in which several water oxygen atoms are within short distances of protein C-H groups. They hypothesized that C-H…O bonds may be important in macromolecules. At the time when this paper was published, X-ray crystallography was experiencing rapid changes with the introduction of X-ray synchrotron sources and imaging plates as well as CCD detectors replacing film, both breakthroughs allowing for high-resolution data collection and accurate refinement of protein atomic models [105]. Intrigued by Steiner and Saenger’s paper, in 1995, we analyzed thirteen crystal structures at a resolution of 1–2 Å and discovered overwhelming stereochemical evidence of C-H…O bonds [106]. Arguably, the most dramatic case was that of the Cα-H…O=C bonds, as predicted by Huggins [93], whose paper was regrettably unknown to us at the time. Specifically, we demonstrated that close Cα-H…O=C interactions, with stereochemistry consistent with H-bond character, are common across the strands in both parallel and antiparallel β-sheets, thus engaging the second lone electron *sp^2^* pair (the first acts as an acceptor in the canonical N-H…O=C inter-strand bond). This was reaffirmed soon afterward by other authors using a different set of crystal structures, and it was additionally noted, not unexpectedly, that Gly—recurrent in β-sheets—is not a donor in these interactions [107]. Shortly thereafter, Bella and Berman described the precise stereochemistry of Cα-H…O=C interactions in collagen, based on their 1.85 Å resolution structure [108] (Figure 3). The stereochemistry of these bonds again aligns perfectly with the one proposed by Huggins, although the authors also appear to have been unaware of Huggins’s publication.

**Figure 4:**
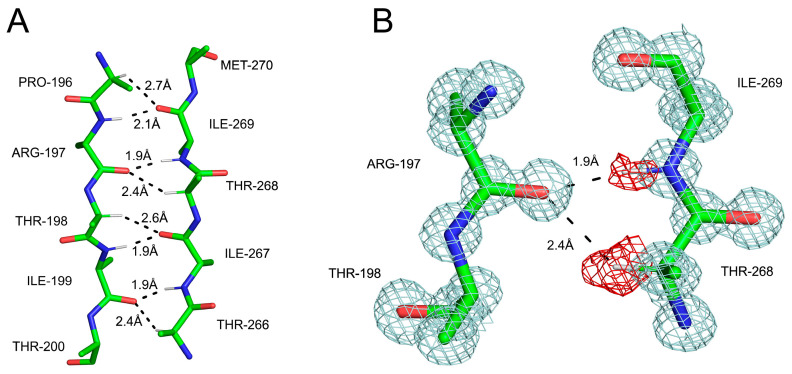
The two types of hydrogen bonds, donated, respectively, by amide and Cα-bonded protons to carbonyl oxygens in an antiparallel β-sheet structure in the PDZ2 domain of syntenin [110], visualized at 0.73 Å resolution: (**A**) an atomic model showing two backbone fragments with interstrand H-bonds and their lengths shown; (**B**) electron density (2Fobs-Fcalc and Fobs-Fcalc) around one specific pair of H-bonds showing the positive difference density associated with hydrogen atoms.

Many high-resolution protein structures have since confirmed our early results. The precise stereochemistry of these bonds was validated through refinement at subatomic resolution, including our own work on the PDZ2 domain of syntenin at 0.73 Å resolution [110] (Figure 4), or the structure of myelin protein 2 at 0.72 Å resolution [111]. One of these studies demonstrated that the positions of the hydrogen atoms bound to Cα deviate by about 0.2 to 0.3 Å from idealized *sp^3^* C-H geometries, with the hydrogen being bent toward the oxygen acceptor [112]. It is now recognized that the Cα-H group in peptides is polarized and relatively acidic. Ab initio calculations using N,N-dimethylformamide as a model system showed that the energy of the Cα-H…O=C bond is 4.0 ± 0.5 kcal/mol [113]. This was subsequently revised for isolated amino acids to be between 1.9 and 2.5 kcal/mol [114]. Owing to the excellent studies by Steve Scheiner, an expert in the computation chemistry of H-bonds, we now know that, in the context of β-sheets, there is little difference between the canonical N-H…O=C bonds and those involving Cα-H groups, so the latter types significantly contribute to the stability of the β-sheet secondary structure [115,116].

A direct experimental observation of the Cα-H…O=C bonds in proteins was reported in 2003 by Cordier et al. [117], who made the first NMR observation of H-bond scalar coupling ^h3^J_CαC′_ correlations across these bonds in the β-sheet regions of an immunoglobin binding domain of protein G.

In α-helices, Cα-H groups do not participate in any regular network but do occur within specific motifs [118]. They have been found in helix-terminating motifs, especially X-Gly and X-non-Gly, and as a part of the helix-stopping Schellman motif [118]. However, the stereochemistry of these interactions is less favorable than that of β-sheets, and it is uncertain if they are uniformly energetically significant. There is, nonetheless, one specific case in which Cα-H groups have an important role: the stabilization of helical bundles in membrane proteins through interhelical, i.e., Cα-H…O=C, backbone-to-backbone contacts [119,120,121].

Finally, aside from regular secondary structure elements, Cα-H-mediated H-bonds were also found to stabilize specific turns [122]. Interestingly, in a number of cases where buried Cα-H are not engaged in an H-bond enforced by the secondary structure, they often still donate to side-chain acceptors, such as in the penicillin-binding protein 2a [123], where His143 Cα-H is within 2.5 Å of a capping side-chain carbonyl of Asn307 (Figure 4).

To conclude, Cα-H…O bonds are now considered as one of the principal secondary forces in protein folding [124].

#### 3.2.2. Bonds Involving C-H Methine Groups in His

Histidine contains two potent H-bond donor groups: Cε-H and Cδ-H. Their character is contingent on the tautomer; that is, the hydrogen/tritium exchange rate is two orders of magnitude higher for Cε-H for protonated, in comparison to neutral His [125]. An ab initio computational study showed that neutral histidine binds a water molecule at Cε-H and Cδ-H with 2.4 and 2.3 kcal/mol, respectively, while for protonated histidine, the corresponding values are 11.3 and 9.5 kcal/mol [126]. These are by far the strongest interactions involving C-H donor groups in proteins, exceeding twice the energy of a canonical H-bond. The corresponding C-H distances are predicted to lengthen by 7.8 and 5.7 mÅ, producing a redshift in the stretching frequency [126]. In agreement with these results, data mining in high-resolution protein crystal structures revealed that His Cε-H and Cδ-H groups show distinct bias toward linearity [127].

**Figure 5:**
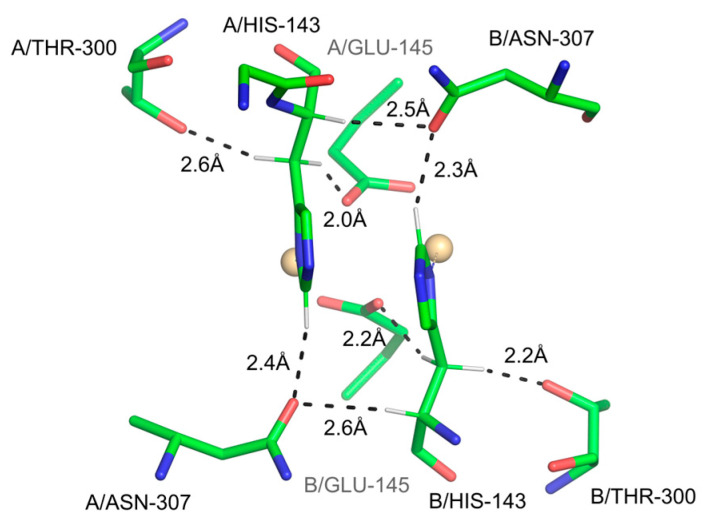
A constellation of amino acids at the interface of a homodimer in the penicillin-binding protein 2a (PDB code 3ZFZ, resolution 2.25 Å). Note how the Cα-H of His143 is capped by the side-chain carbonyl of Asn 307, which also accepts an H-bond from the Cε-H group of His143 across the interface. In addition, the two Cβ-H hydrogen atoms appear to be donating bonds to two side-chain oxygen atoms, as inferred from short distances and stereochemistry. The two spheres are Cd^2+^ ions.

**Figure 6:**
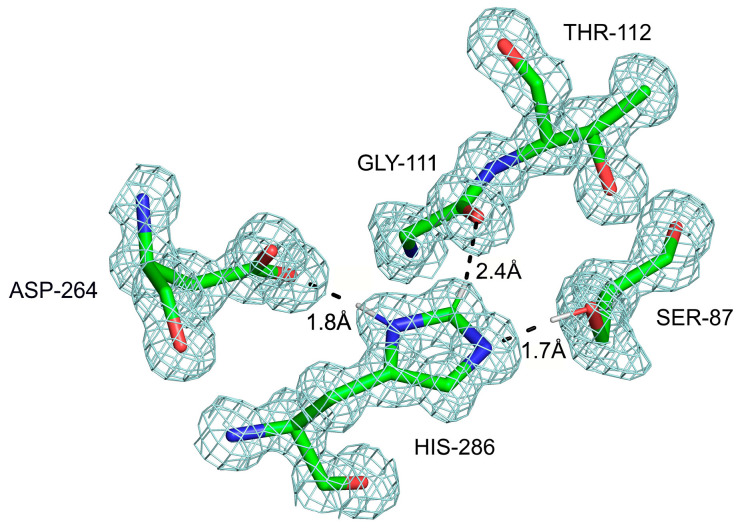
The catalytic center (also known as the catalytic Asp-His-Ser triad) in one of the serine hydrolases from the α/β hydrolase superfamily, with a crystal structure solved to 1.08 Å resolution (PDB code 7COF) [131]. Three hydrogen bonds are shown, including the Cε-H…O=C between the catalytic histidine and a backbone carbonyl oxygen of Gly111. The dihedral angle ^Gly111^Cα-C–O…H is 16.2°, while the angle C=O…H is 144.1°, placing the proton very near the *sp^2^* plane of the carbonyl oxygen.

A survey of the structures obtained from both neutron and X-ray diffraction reveals a multitude of C-H…O interactions involving the imidazole ring [127,128] (e.g., Figure 5). Their strength suggests functional implications. In fact, the first observation of a very close Cε-H…O=C contact was reported by our laboratory for the active site serine hydrolases, regardless of the tertiary fold [129] (Figure 6). This led to proposals for a revision of the catalytic mechanism in this family of enzymes [130]. A range of enzymes contains histidines within their active sites, and the C-H groups of imidazole are involved in various interactions. For example, in carbonic anhydrase, three histidines bind Zn^2+^, and the conformation of one is stabilized by a Cε-H acting as a donor to carboxyl of Glu106 [128]. No comprehensive survey of thefunctional roles of C-H…O bonds in enzymatic reactions has been published to date.

#### 3.2.3. Bonds Involving Cδ1-H Group from Trp

**Figure 7:**
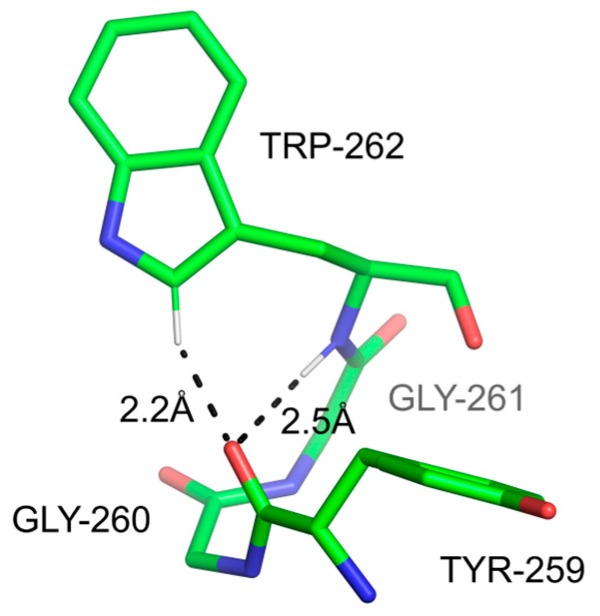
A hydrogen bond involving the Cδ-H donor group from Trp259 in S-adenosylmetione synthase (PDB code 6VCW [133]). The bond caps a free main chain carbonyl oxygen at the break of an α-helix. Note that the carbonyl accepts two H-bonds via its *sp^2^* electron pairs.

The Cδ1-H group of Trp, located next to N within the five-member ring of imidazole, is another potential strong donor for H-bonds. Computational studies showed that the energy of binding of this group to a water molecule is 2.1 kcal/mol, albeit with marginal change to the C-H bond length and no stretching frequency shift [126]. C-H-mediated H-bonds, including the Cδ1-H group but also involving the *sp^3^* Cβ-H, have been recognized as essential for the stabilization of frequently occurring non-canonical conformations of Trp [132]. In contrast, a subsequent study of aromatic carbon H-bond donor groups concluded that the Cδ1-H group shows no bias toward linearity with acceptor moieties, effectively discounting its role as a good H-bond donor [127]. Our recent study (Szczygiel, M.; Minor, W.; and Derewenda, Z.S.; manuscript in preparation) uncovered numerous examples of specific turns and secondary structure motifs clearly stabilized by Trp Cδ-H…O=C H-bonds (Figure 7).

#### 3.2.4. The *sp^3^* C-H Groups

In our original report of C-H…O H-bonds in proteins, we have also presented examples of such interactions between the main chain carbonyl groups in α-helices and Cβ/Cγ bonded hydrogens within the next turn of the helix [106]. The suggestion that these are cohesive interactions has been also reiterated by other authors [111,118], although such inferences based on purely stereochemical arguments, involving weakly polarized C-H bonds, have to be taken with caution.

**Figure 8:**
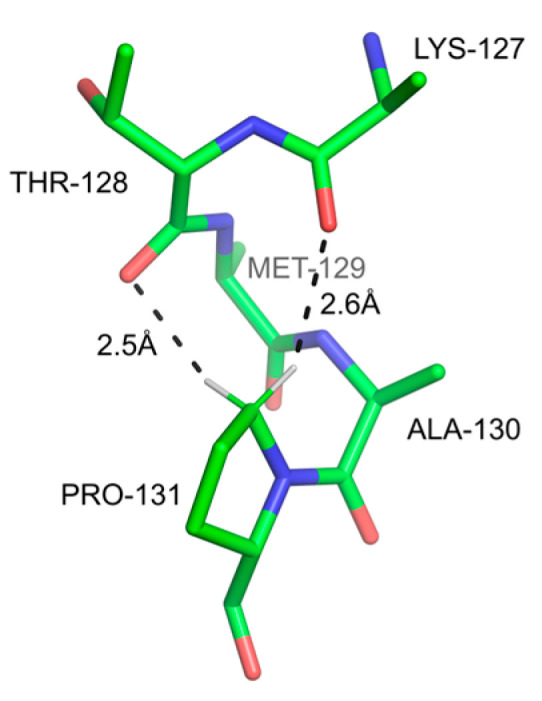
H-bonds by two Cδ-H hydrogen atoms of Pro131 in the structure of sarcoplasmic Ca^2+^-binding protein (PSB code 2SCP [139]). It has been shown through theoretical calculations that this is a cohesive interaction with a significant free-energy contribution [138].

One case of *sp^3^* C-H groups involves prolines [134]. This is particularly interesting because Pro is a unique amino acid lacking a hydrogen-bonding amide group and therefore strongly disrupting both α- and β-sheet secondary structures. The pyrrolidine ring contains two *sp^3^* carbon groups (Cδ-H, and Cα-H), which are polarized by the adjacent electron-withdrawing nitrogen. The polarization was evidenced by NMR downfield chemical shifts, which are greater in magnitude than aliphatic groups [135,136,137]. An early survey of protein crystal structures revealed that, when Pro occurs in α-helices, the canonical H-bond is often replaced by a Cδ-H-mediated close contact, with carbonyl oxygen atoms in the preceding turn of the helix (3–4 residues upstream). Interestingly, specific conformers of the pyrrolidine ring were contingent on the presence of these bonds [134]. The proximity of *sp^3^* C-H and carbonyl groups was suggestive but not conclusive with respect to energetic favorability. Subsequent computation studies suggested that the energetic consequences were dependent on structural context: they appeared not to be stabilizing, in cases where they preceded a canonical peptide H-bond between (i + 1) N-H and (i − 3) C=O, and generally stabilizing in the absence of such peptide bond [138] (Figure 8).

The question of the H-bonds mediated by the *sp^3^* C-H groups in proline was revisited recently. A series of three Pro derivatives were synthesized and studied in a crystalline state to visualize the intermolecular C-H…O contacts. Furthermore, analogous interactions were analyzed in 824 crystal structures of other derivatives from the Cambridge Structural Database [140]. The close contacts in crystal structures were found to be ubiquitous, and—importantly—were not restricted to Cδ-H and Cα-H, as since Cβ-H and Cδ-H were also involved in close contacts. This detailed study demonstrated the ubiquity of C-H…O interactions involving Pro.

#### 3.2.5. Protein–Ligand Interactions

Following the discoveries of C-H-mediated intramolecular bonds in proteins, it has also been observed that such bonds are found and may be significant in intermolecular interactions involving protein–protein [141] and protein–ligand interfaces [142,143]. Here, we use the term ‘ligand’ in the broadest possible sense, i.e., any small organic molecule including enzyme substrates, products, cofactors, inhibitors, etc. Many natural compounds occurring in human bodies, such as nucleotides, cofactors, vitamins, etc., include heterocyclic rings with polarized methine or *sp^3^* C-H groups, so it is not surprising that these groups may serve as additional anchors in protein–ligand binding.

**Figure 9:**
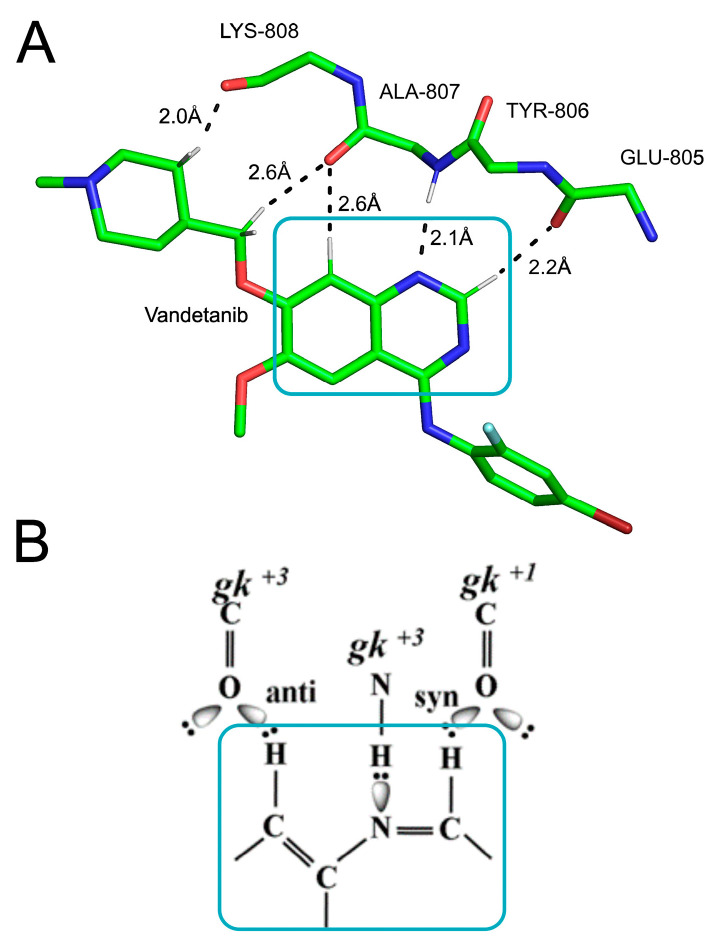
The binding of a representative FDA-approved drug, vandetanib, to the target RET kinase [148]: (**A**) a structural diagram depicting four C-H…O bonds donated by the drug compound; (**B**) one of the primary three scaffolds, identified by Derewenda et al. [146] among FDA-approved drugs, that target the hinge portion of the kinase with two C-H…O bonds.

Among the cases that attracted considerable attention owing to clinical implications are protein kinases, which are well-established drug targets, commonly in cancer therapy [144,145]. Kinases use a distinct oligopeptide hinge motif with two solvent-exposed main-chain carbonyl groups to engage ATP, its cofactor, through H-bonds accepted from the N10- and C(2)-bound protons in adenine [146]. In fact, together with the backbone amide—located between the two solvent-exposed carbonyl oxygens—donating an H-bond to N1 of adenine, the three interactions ensure the recognition of adenine in the binding pocket, although often the C(2) methine group is ignored in the literature [147]. As heterocyclic, aromatic moieties are ubiquitous among drug molecules targeting kinases in various cancers, it is not surprising to see the carbonyl groups of the hinge accept H-bonds from various methine C-H groups of these compounds. A recent comprehensive analysis revealed that most FDA-approved kinase inhibitors in fact harbor a limited set of scaffolds that saturate the H-bonding potential of the hinge with highly polarized methine groups [146] (Figure 9).

**Figure 10:**
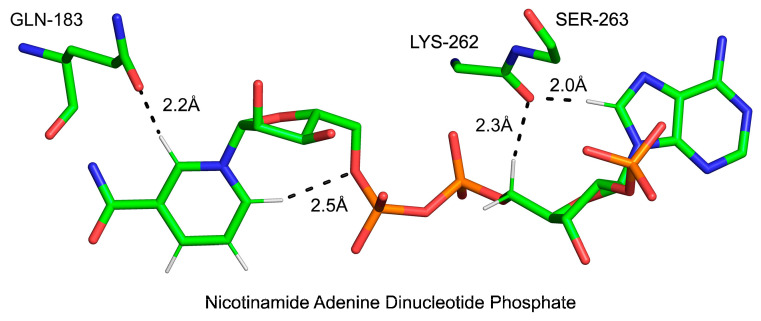
Select details of the interaction of NADP^+^ with human aldose reductase. Only residues accepting C-H bonds from the dinucleotide are shown for clarity. Note the two strong bonds donated by C(8)-H of adenine and C(5)′-H of ribose to the backbone carbonyl of Lys262, as well as in the intramolecular bond between C(6)-H of nicotinamide and O(5) of adjacent ribose, as well as C(2)-H of nicotinamide and the side-chain carbonyl of Gln183. Only relevant hydrogen atoms and those on the nicotinamide are shown.

The field of C-H…O bonds in protein–ligand interactions has not been well explored. In an ongoing project in our laboratory, we have identified many examples where such bonds appear to play a crucial role (Derewenda Z.S. and Derewenda U.; manuscript in preparation). One of the examples from a recent study is the binding of the cofactor (S-adenosyl-L-methionine) by a viral methyl transferase [149]. Another interesting example we identified is the interaction of the nicotinamide adenine dinucleotide phosphate (NADP^+^) with human aldose reductase, visualized with a crystal structure determined with high precision at 0.76 Å resolution [150] (PDB code 4LBS, see Figure 10 for details). Clearly, the role of C-H…X bonds in protein–ligand interactions is yet to be fully explored.

### 3.3. Nucleic Acids

DNA and RNA contain two purine bases, i.e., adenine (A) and guanine (G), and three pyrimidine bases, i.e., cytosine (C), the DNA specific thymine (T), and RNA-specific uracil (U), covalently linked to a phosphate–pentose (ribose or deoxyribose) backbone. There are only a few polarized methine groups capable of acting as H-bond donors: C(8)-H in both A and G, C(2)-H in G, C(6)-H and C(5)-H in U and C, and C(6) in thymine. As we have already seen, the C(6) in pyrimidines and C(8) in purines were identified very early on as H-bond donors in the interaction with the sugar O(5)’. However, it is important to stress that C-H…O bonds are ubiquitous and play an important role in base pairing, as well as base–backbone interactions. There are, of course, *sp^3^* C-H groups within the pentose. All these chemical moieties may be engaged in H-bonds in several ways.

#### 3.3.1. C-H…O Bonds in Base Pairing

The notion that canonical H-bonds (i.e., between electronegative groups) may play a role in DNA structure was first put forward based on biophysical studies of DNA in solution in the 1940s [151,152]. However, it was Watson and Crick who first proposed that these bonds are responsible for the complementary association of two antiparallel strands of DNA through the specific pairing of GC and AT [81,153]. Famously, they assigned only two H-bonds in the GC pair, because the imprecise models they used slightly distorted the geometry of the third putative H-bond, N(2)-H^guanine^…O(2)^cytosine^, which was thus erroneously ruled out by Jerry Donohue [154]. This was corrected by Pauling and Corey in 1956 [155] but was only visualized in 1963, with the solution of the crystal structure of 9-thylguanine complexed with 1-methylcytosine [156]. The presence of three H-bonds was in agreement with the enhanced stability of the CG pairs as compared to AT(U). Surprisingly, however, the first single-crystal, high-resolution X-ray diffraction study of an analog of the AT pair (a complex of 9-methyladenine and 1-methylthymine, in which the nitrogen atoms bound to the pentose were blocked through methylation) revealed an alternative hydrogen bond pattern, known from the name of the author of the study as Hoogsteen pairing [157]. In this structure, adenine is flipped upside down, which in the context of a nucleoside is equivalent to a 180° rotation of the adenine around the N9-C1′ glycosidic bond. Surprisingly, such pairing was previously proposed theoretically to explain the formation of triplexes of RNA [158]. Spectroscopic evidence also suggested that Hoogsteen-type pairing may be found in GC pairs, with identical flip of guanine, although this stereochemistry required the protonation of cytosine [159]. The existence of a Hoogsteen GC pair was visualized years later using crystallography [160].

**Figure 11:**
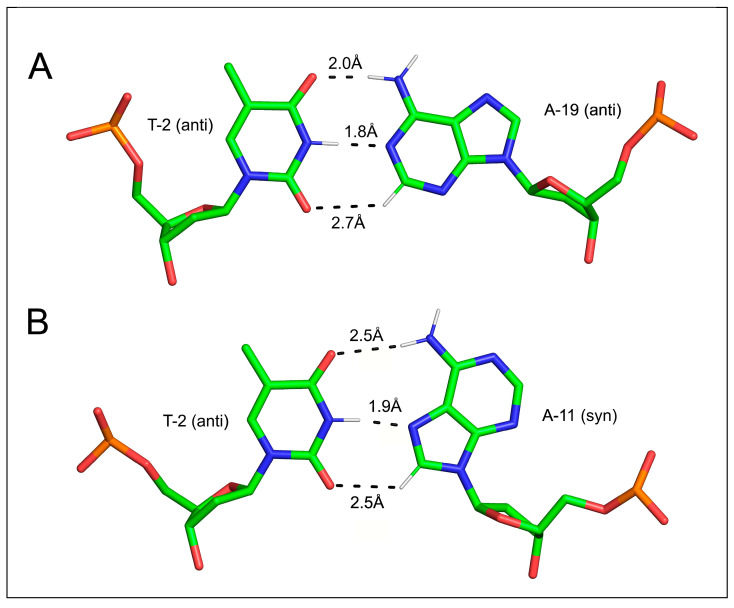
The thymine–adenine base pairing in DNA: (**A**) Watson–Crick and (**B**) Hoogsteen pairing. C(2)H^adenine^…O(2)^thymine^ in the Watson–Crick pair and C(8)H^adenine^…O(2)^thymine^ in the Hoogsteen pair.

In 1996, Hunter and colleagues first noted that the Watson–Crick AT base pair contains a close contact that is topologically similar to the third bond in the GC pair, i.e., C(2)H^adenine^…O(2)^thymine^, and suggested that a similar type of interaction contributes to the stability of the Hoogsteen AT base pair [161] (Figure 11). Contemporaneous theoretical studies predicted that this contributes 6% of the binding energy in the pair [162]. Nevertheless, the existence of this H-bond remained controversial, as other authors maintained that it is very weak [163] or even entirely of a van der Waals nature [164].

However, supporting data kept emerging, from spectroscopy as well as computational approaches [165,166,167,168,169,170]. The most recent of these studies [168,169,170] strongly reaffirmed the significance of C-H…O(N) bonds in base pairing in both natural and unnatural base pairs, especially in the Watson–Crick and Hoogsteen AT(U) pairs. They revealed in particular that the chemical context of DNA strengthens the C-H…O bond while decreasing the energy of the N-H…O bond [170].

Interestingly, recent research on DNA introduced a range of unnatural or non-Watson–Crick base pairs, which include artificial chemistry replacing conventional bases, sugars, and the phosphate backbone. Many of such unnatural base pairs were also found to contain C-H…O bonds stabilizing their interactions [170,171,172].

Although most of the above studies were focused on both DNA and RNA, specific investigations of RNA have also revealed the importance of C-H…O bonds in base pairing [173].

#### 3.3.2. C-H…O Base–Backbone Bonds in NA

As the number of known crystal structures of nucleosides and oligonucleotides increased, they revealed the rich spectrum of conformation in DNA and RNA, the latter being particularly diverse [174,175,176]. There are two main conformation subfamilies in each of the three types of DNA, namely A, B, and Z, which are denoted as A1, A2, B1, B2, Z1, and Z2 [177,178]. The difference stems from two sets of dihedral angles around the P-O3′-C3′ bond. This diversity can be traced to the flexibility in the stereochemistry of the individual nucleosides. Arguably, one of the best computational analyses of such an isolated nucleoside, namely thymidine, was conducted by Yurenko et al. [179], who took into account all 92 conformers. They observed that all major conformers had the typical C(6)-H…O5′ bond, but the B2 and A2 conformers had a bifurcated bond such that C(6)-H was a donor to O4′ as well. The same authors later expanded their analysis to all A1 and B1 conformers [166], confirming the ubiquity of interaction and calculating their energies, which, depending on the nucleoside and conformer, fell in the range of 1–5 kcal/mol [166].

Base-to-backbone C-H…O bonds also occur in contexts other than A- or B-DNA. For example, NMR and X-ray diffraction studies of four stranded intercalated cytosine-rich structures revealed an intra-cytidine bond between C(6)H and the β-lone electron pair of the O4′ within deoxyribose [180]. Crystallographic and molecular dynamics studies of the anticodon hairpin of tRNA^Asp^ confirmed the ubiquitous nature of the C(6/8)-H…O5′ hydrogen bonds in tRNA [181], and other studies expanded this to RNA in general [182]. A more recent study using density functional theory in combination with natural bond orbital analysis provided in-depth insights into the role of these interactions in tRNA anticodon structures [183].

## 4. Conclusions

Although the notion that C-H groups can serve as donors in hydrogen bonds took nearly five decades to become widely accepted, it is very clear today that these interactions play significant roles in organic compounds, especially in proteins and nucleic acids. In agreement with theory, polarized C-H groups, and in particular methine groups found in nucleic acid bases, as well as the amino acids histidine and tryptophan, are particularly good donors. However, proteins have one other important donor group, Cα-H, which, in spite of having an *sp^3^* hybridization, is also notably polarized in the context of the adjacent peptide bonds. The latter group contributes significantly to the stability of the β-sheet secondary structure, while histidine is involved in numerous interactions including enzyme active site centers with potential functional implications. It is important to note that, based on the example of protein kinases and their inhibitors, it is clear that C-H-mediated H-bonds play an important role in protein–ligand interactions, which is not yet fully understood but is of critical importance to drug discovery. Although typically referred to as ‘weak’ hydrogen bonds, in proteins, these interactions involving polarized C-H donors form bonds with energies in the ~3–13 kcal/mol range, in some cases rivaling the strength of canonical H-bonds between electronegative atoms.

Finally, it is important to remember that life on Earth evolved owing to the unique structures of DNA and RNA. Several C-H…O H-bonds play key roles in the stabilization of these structures.

## Figures and Tables

**Figure 1:**
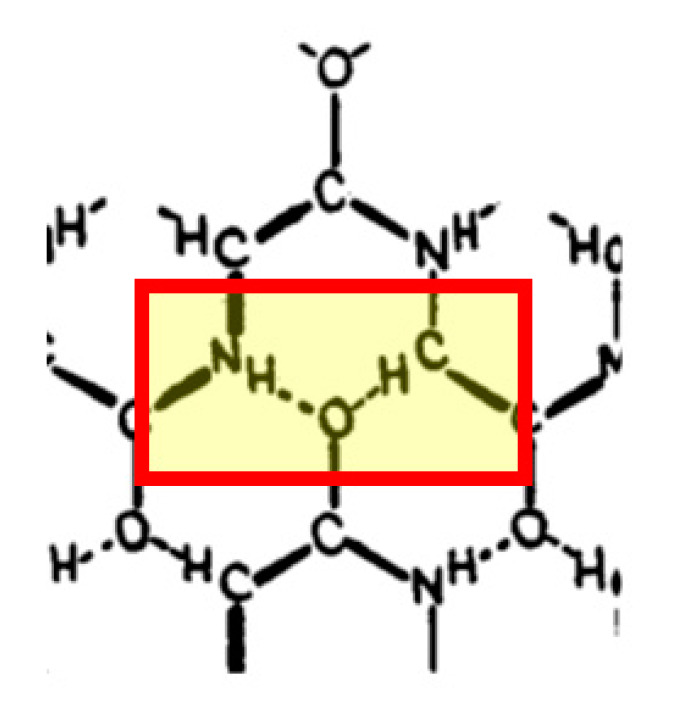
The two H-bonds donated by the amide and Cα bound hydrogens in the atomic model of a layer of collagen proposed by Huggins in 1943 (adapted from Figure 17 of [93]). The red rectangle shows the two H-bonds accepted by a single peptide carbonyl. This is historically the first suggestion of an H-bond mediated by the Cα-H groups in proteins.

## Data Availability

Not applicable.

## References

[1] Latimer W.M., Rodebush W.H. (1920). Polarity and ionization from the standpoint of the Lewis theory of valence. J. Am. Chem. Soc..

[2] Derewenda Z.S. (2021). On the centennials of the discoveries of the hydrogen bond and the structure of the water molecule: The short life and work of Eustace Jean Cuy (1897–1925). Acta Crystallogr. Sect. A Found. Adv..

[3] Weinhold F., Klein R.A. (2012). What is a hydrogen bond? Mutually consistent theoretical and experimental criteria for characterizing H-bonding interactions. Mol. Phys..

[4] Quane D.J. (1990). The Reception of the Concept of Hydrogen-Bonding by the Chemical Community 1920–1937. Bull. Hist. Chem..

[5] Huggins M.L. (1971). 50 Years of Hydrogen Bond Theory. Angew. Chem. Int. Ed..

[6] Huggins M.L. (1980). The Hydrogen-Bond and Other Reminiscences. ChemTech.

[7] Pauling L. (1932). The nature of the chemical bond IV The energy of single bonds and the relative electronegativity of atoms. J. Am. Chem. Soc..

[8] Hildebrand J.H. (1927). A Quantitative Treatment of Deviations from Raoult’s Law. Proc. Natl. Acad. Sci. USA.

[9] Bowler M.G., Bowler D.R., Bowler M.W. (2017). Raoult’s law revisited: Accurately predicting equilibrium relative humidity points for humidity control experiments. J. Appl. Crystallogr..

[10] Dolezalek F. (1908). On the theory of binary mixture and concentrated solutions. Z. Phys. Chem..

[11] Dolezalek F., Schulze A. (1913). The theory of binary mixtures and concentrated solutions. IV. The mixture: Ethyl ether chloroform. Z. Phys. Chem..

[12] Dolezalek F. (1913). The theory of binary mixtures and concentrated solutions. III. Responses to the gentelmen T.S. Patterson and J.J. van Laar. Z. Phys. Chem..

[13] Lewis G.N. (1916). The atom and the molecule. J. Am. Chem. Soc..

[14] Wyatt W.F. (1929). Solutions Part III. The transition point of carbon tetrachloride, and compounds of carbon tetrachloride or chloroform with acetone, ether, and benzene. Trans. Faraday Soc..

[15] Moelwyn-Hughes E.A., Sherman A. (1936). Sow kinetic consequences of complex formation in solution. J. Chem. Soc..

[16] Bernal J.D., Megaw H.D. (1935). The function of hydrogen in intermolecular forces. Proc. R. Soc. Lond. Ser. A.

[17] Glasstone S. (1937). The structure of some molecular complexes in the liquid phase. Trans. Faraday Soc..

[18] Huggins M.L. (1936). Hydrogen bridges in organic compounds. J. Org. Chem..

[19] Zellhoefer G.F. (1937). Solubility of halogenated hydrocarbon refrigerants in organic solvents. Ind. Eng. Chem..

[20] Leonard N.J. (1989). Marvel, Carl, Shipp—September 11, 1894 January 4, 1988. Org. Synth..

[21] Zellhoefer G.F., Copley M.J., Marvel C.S. (1938). Hydrogen bonds involving the C-H link—The solubility of haloforms in donor solvents. J. Am. Chem. Soc..

[22] Zellhoefer G.F., Copley M.J. (1938). The heats of mixing of haloforms and polyethylene glycol ethers. J. Am. Chem. Soc..

[23] Copley M.J., Zellhoefer G.F., Marvel C.S. (1938). Hydrogen bonds involving the C-H link IV The effect of solvent association on solubility. J. Am. Chem. Soc..

[24] Copley M.J., Zellhoefer G.F., Marvel C.S. (1938). Hydrogen bonds involving the C-H link V The solubility of methylene chloride in donor solvents. J. Am. Chem. Soc..

[25] Copley M.J., Holley C.E. (1939). Hydrogen bonding by negatively substituted CH groups. VI. Acetylenic compounds. J. Am. Chem. Soc..

[26] Copley M.J., Marvel C.S., Ginsberg E. (1939). Hydrogen Bonding by S-H. VII. Aryl Mercaptans. J. Am. Chem. Soc..

[27] Copley M.J., Zellhoefer G.F., Marvel C.S. (1939). Hydrogen Bonds Involving the C-H Link. VIII. The Solubilities of Completely Halogenated Methanes in Organic Solvents. J. Am. Chem. Soc..

[28] Copley M.J., Zellhoefer G.F., Marvel C.S. (1940). Hydrogen bonds involving the C-H link IX Nitriles and dinitriles as solvents for hydrogen containing halogenated methanes. J. Am. Chem. Soc..

[29] Marvel C.S., Dietz F.C., Copley M.J. (1940). Hydrogen bonds involving the C-H link. X. The solubility of donor solutes in halogenated hydrocarbons. J. Am. Chem. Soc..

[30] Marvel C.S., Copley M.J., Ginsberg E. (1940). Hydrogen bonds involving the C-H link. XI. Effect of structure on bonding of donor and acceptor molecules. J. Am. Chem. Soc..

[31] Marvel C.S., Copley M.J., Ginsberg E. (1940). Hydrogen bonds involving the C-H <- F link. XII. J. Am. Chem. Soc..

[32] Copley M.J., Ginsberg E., Zellhoefer G.F., Marvel C.S. (1941). Hydrogen Bonding and the Solubility of Alcohols and Amines in Organic Solvents. XIII. J. Am. Chem. Soc..

[33] Marvel S.C., Harkema J., Copley M.J. (1941). Hydrogen Bonds Involving the C-H Link. XIV. Solubility of Donor Solutes in Hydrogen Bonding Solvents. J. Am. Chem. Soc..

[34] Marvel C.S., Harkema J. (1941). Hydrogen bonds involving the C-H link. XV. Non-bonding of triphenylmethane hydrogen atoms. J. Am. Chem. Soc..

[35] Hunter L. (1946). The hydrogen bond. Ann. Rep. Progr. Chem..

[36] Gordy W. (1938). Association of unlike molecules through hydrogen bonds. Nature.

[37] Gordy W. (1938). Infrared absorption studies of hydrogen bonds between unlike molecules. J. Am. Chem. Soc..

[38] Gordy W. (1939). Spectroscopic evidence of hydrogen bonds: Chloroform and bromoform in donor solvents. J. Chem. Phys..

[39] Klemperer W., Cronyn M.W., Maki A.H., Pimentel G.C. (1954). Infrared Studies of the Association of Secondary Amides in Various Solvents. J. Am. Chem. Soc..

[40] Huggins C.M., Pimentel G.C., Shoolery J.N. (1955). Proton Magnetic Resonance Studies of Chloroform in Solution—Evidence for Hydrogen Bonding. J. Chem. Phys..

[41] Pimentel G.C., McClellan A.L. (1960). The Hydrogen Bond.

[42] Campbell A.N., Kartzmark E.M. (1960). The Energy of Hydrogen Bonding in the System—Acetone-Chloroform. Can. J. Chem..

[43] Allerhand A., Schleyer P.V. (1963). A Survey of C-H Groups as Proton Donors in Hydrogen Bonding. J. Am. Chem. Soc..

[44] Dulmage W.J., Lipscomb W.N. (1951). The Crystal Structures of Hydrogen Cyanide, Hcn. Acta Crystallogr..

[45] Sutor D.J. (1963). Crystal and Molecular Structure of 1,3,7,9-Tetramethyluric Acid. Acta Crystallogr..

[46] Sutor D.J. (1962). C-H⋯O Hydrogen Bond in Crystals. Nature.

[47] Sutor D.J. (1963). Evidence for Existence of C-H⋯O Hydrogen Bonds in Crystals. J. Chem. Soc..

[48] Furberg S. (1950). The Crystal Structure of Cytidine. Acta Crystallogr..

[49] Shefter E., Trueblood K.N. (1965). Crystal and Molecular Structure of D(Plus)-Barium Uridine-5′-Phosphate. Acta Crystallogr..

[50] Sharma B.D., Mcconnell J.F. (1965). Crystal and Molecular Structure of Isocytosine. Acta Crystallogr..

[51] Sundaralingam M. (1966). Stereochemistry of Nucleic Acid Constituents. III. Crystal and Molecular Structure of Adenosine 3′-Phosphate Dihydrate (Adenylic Acid B). Acta Crystallogr..

[52] Haschemeyer A.E., Rich A. (1967). Nucleoside Confromations—An Analysis of Steric Barriers to Rotation About Gylcosidic Bond. J. Mol. Biol..

[53] Donohue J. (1952). The Hydrogen Bond in Organic Crystals. J. Phys. Chem..

[54] Ramachandran G.N., Ramakrishnan C., Sasisekharan V. (1963). Stereochemistry of polypeptide chain configurations. J. Mol. Biol..

[55] Schwalbe C.H. (2012). June Sutor and the C-H⋯O hydrogen bonding controversy. Crystallogr. Rev..

[56] Desiraju G.R. (1991). The C-H⋯O Hydrogen-Bond in Crystals—What Is It. Acc. Chem. Res..

[57] Olympia P.L. (1970). Some theoretical aspects of C-H⋯O bonding. Chem. Phys. Lett..

[58] Green R.D. (1974). Hydrogen Bonding by C-H Groups.

[59] Kollman P., Mckelvey J., Johansson A., Rothenberg S. (1975). Theoretical Studies of Hydrogen-Bonded Dimers—Complexes Involving Hf, H2o, Nh3, Hcl, H2s, Ph3, Hcn, Hnc, Hcp, Ch2nh, H2cs, H2co, Ch4, Cf3h, C2h2, C2h4, C6h6, F-, and H3o+. J. Am. Chem. Soc..

[60] Gay R., Vanderkooi G. (1981). Hydrogen-Bonding of Phosphodiesters to Water, Methanol, and Methylamine as Studied by the Cndo-2 Method. J. Chem. Phys..

[61] Umeyama H., Morokuma K. (1977). Origin of Hydrogen-Bonding—Energy Decomposition Study. J. Am. Chem. Soc..

[62] Taylor R., Kennard O. (1982). Crystallographic Evidence for the Existence of C-H⋯O, C-H⋯N, and C-H⋯C1 Hydrogen-Bonds. J. Am. Chem. Soc..

[63] Sarma J.A.R.P., Desiraju G.R. (1986). The Role of Cl=Cl and C-H=O Interactions in the Crystal Engineering of 4-a Short-Axis Structures. Acc. Chem. Res..

[64] Sarma J.A.R.P., Desiraju G.R. (1987). C-H⋯O Interactions and the Adoption of 4-a Short-Axis Crystal-Structures by Oxygenated Aromatic-Compounds. J. Chem. Soc. Perkin Trans..

[65] Desiraju G.R. (2013). Crystal Engineering: From Molecule to Crystal. J. Am. Chem. Soc..

[66] Desiraju G.R. (2011). Crystal engineering. From molecules to crystals. Acta Crystallogr. Sect. A Found. Adv..

[67] Budesinsky M., Fiedler P., Arnold Z. (1989). Triformylmethane—An Efficient Preparation, Some Derivatives, and Spectra. Synthesis.

[68] Boldeskul I.E., Tsymbal I.F., Ryltsev E.V., Latajka Z., Barnes A.J. (1997). Reversal of the usual nu(C-H/D) spectral shift of haloforms in some hydrogen-bonded complexes. J. Mol. Struct..

[69] Hobza P., Havlas Z. (2000). Blue-shifting hydrogen bonds. Chem. Rev..

[70] Alabugin I.V., Manoharan M., Peabody S., Weinhold F. (2003). Electronic basis of improper hydrogen bonding: A subtle balance of hyperconjugation and rehybridization. J. Am. Chem. Soc..

[71] Joseph J., Jemmis E.D. (2007). Red-, blue-, or no-shift in hydrogen bonds: A unified explanation. J. Am. Chem. Soc..

[72] Scheiner S., Kar T. (2008). Spectroscopic and structural signature of the CH-O hydrogen bond. J. Phys. Chem. A.

[73] Arunan E., Desiraju G.R., Klein R.A., Sadlej J., Scheiner S., Alkorta I., Clary D.C., Crabtree R.H., Dannenberg J.J., Hobza P. (2011). Definition of the hydrogen bond (IUPAC Recommendations 2011). Pure Appl. Chem..

[74] Vaz P.D., Nolasco M.M., Gil F.P.S.C., Ribeiro-Claro P.J.A., Tomikinson J. (2010). Hydrogen-Bond Dynamics of C-H⋯O Interactions: The Chloroform⋯Acetone Case. Chem.-Eur. J..

[75] Kankanamge S.R.G., Ma J.B., Mackin R.T., Leonik F.M., Taylor C.M., Rubtsov I.V., Kuroda D.G. (2020). Proving and Probing the Presence of the Elusive C-H⋯O Hydrogen Bond in Liquid Solutions at Room Temperature. Angew. Chem. Int. Ed..

[76] Pullanchery S., Kulik S., Rehl B., Hassanali A., Roke S. (2021). Charge transfer across C-H⋯O hydrogen bonds stabilizes oil droplets in water. Science.

[77] Yu Y.Q., Fan W., Wang Y.X., Zhou X.G., Sun J., Liu S.L. (2017). C-H⋯O Interaction in Methanol-Water Solution Revealed from Raman Spectroscopy and Theoretical Calculations. J. Phys. Chem. B.

[78] Shi L.X., Min W. (2023). Vibrational Solvatochromism Study of the C-H···O Improper Hydrogen Bond. J. Phys. Chem. B.

[79] Adhav V.A., Saikrishnan K. (2023). The Realm of Unconventional Noncovalent Interactions in Proteins: Their Significance in Structure and Function. Acs Omega.

[80] Danovich D., Shaik S., Neese F., Echeverria J., Aullon G., Alvarez S. (2013). Understanding the Nature of the CH⋯HC Interactions in Alkanes. J. Chem. Theory Comput..

[81] Watson J.D., Crick F.H. (1953). Molecular structure of nucleic acids; a structure for deoxyribose nucleic acid. Nature.

[82] Franklin R.E., Gosling R.G. (1953). Molecular Configuration in Sodium Thymonucleate. Nature.

[83] Wilkins M.H.F., Stokes A.R., Wilson H.R. (1953). Molecular Structure of Deoxypentose Nucleic Acids. Nature.

[84] Wing R., Drew H., Takano T., Broka C., Tanaka S., Itakura K., Dickerson R.E. (1980). Crystal structure analysis of a complete turn of B-DNA. Nature.

[85] Kypr J., Vorlickova M. (1985). Experiment acknowledged the Watson-Crick hypothesis: A review of B-DNA double helix structural features at atomic resolution. Gen. Physiol. Biophys..

[86] Bernal J.D., Crowfoot D. (1934). X-ray photographs of crystalline pepsin. Nature.

[87] Astbury W.T., Atkin W.R. (1933). X-ray interpretation of the molecular structure of gelatine. Nature.

[88] Kendrew J.C., Bodo G., Dintzis H.M., Parrish R.G., Wyckoff H., Phillips D.C. (1958). A three-dimensional model of the myoglobin molecule obtained by X-ray analysis. Nature.

[89] Pauling L., Corey R.B. (1953). Compound Helical Configurations of Polypeptide Chains—Structure of Proteins of the Alpha-Keratin Type. Nature.

[90] Pauling L., Corey R.B. (1951). The Structure of Hair, Muscle, and Related Proteins. Proc. Natl. Acad. Sci. USA.

[91] Pauling L., Corey R.B. (1951). The Structure of Fibrous Proteins of the Collagen-Gelatin Group. Proc. Natl. Acad. Sci. USA.

[92] Pauling L., Corey R.B., Branson H.R. (1951). The Structure of Proteins—2 Hydrogen-Bonded Helical Configurations of the Polypeptide Chain. Proc. Natl. Acad. Sci. USA.

[93] Huggins M.L. (1943). The structure of fibrous proteins. Chem. Rev..

[94] Furberg S. (1950). An X-ray Study of the Stereochemistry of the Nucleosides. Acta Chem. Scand.

[95] Wilds C.J., Wawrzak Z., Krishnamurthy R., Eschenmoser A., Egli M. (2002). Crystal structure of a B-form DNA duplex containing (L)-alpha-threofuranosyl (3′ -> 2′) nucleosides: A four-carbon sugar is easily accommodated into the backbone of DNA. J. Am. Chem. Soc..

[96] Crick F.H.C., Watson J.D. (1954). The Complementary Structure of Deoxyribonucleic Acid. Proc. R. Soc. Lond. Ser.-A.

[97] Shefter E., Barlow M., Sparks R.A., Trueblood K.N. (1969). Crystal and Molecular Structure of a Dinucleoside Phosphate—Beta-Adenosine-2′-Beta-Uridine-5′-Phosphoric Acid. Acta Crystallogr. Sect. B Struct. Cryst. Crystal Chem..

[98] Seeman N.C., Sussman J.L., Berman H.N., Kim S.H. (1971). Nucleic acid conformation: Crystal structure of a naturally occurring dinucleoside phosphate (UpA). Nat. New Biol..

[99] Saenger W. (1973). Structure and function of nucleosides and nucleotides. Angew. Chem. Int. Ed. Engl..

[100] Jack A., Ladner J.E., Klug A. (1976). Crystallographic refinement of yeast phenylalanine transfer RNA at 2–5A resolution. J. Mol. Biol..

[101] Dragelj J.L., Stankovic I.M., Bozinovski D.M., Meyer T., Veljkovic D.Z., Medakovic V.B., Knapp E.W., Zaric S.D. (2016). C-H/O Interactions of Aromatic CH Donors within Proteins: A Crystallographic Study. Cryst. Growth Des..

[102] Scheiner S. (2010). Theoretical Analysis of the Contributions Made by CH··OH Bonds to Protein Structure. Curr. Org. Chem..

[103] Steiner T., Saenger W. (1993). Role of C-H⋯O Hydrogen-Bonds in the Coordination of Water-Molecules—Analysis of Neutron-Diffraction Data. J. Am. Chem. Soc..

[104] Baker E.N. (1980). Structure of actinidin, after refinement at 1.7 A resolution. J. Mol. Biol..

[105] Walter R.L., Thiel D.J., Barna S.L., Tate M.W., Wall M.E., Eikenberry E.F., Gruner S.M., Ealick S.E. (1995). High-resolution macromolecular structure determination using CCD detectors and synchrotron radiation. Structure.

[106] Derewenda Z.S., Lee L., Derewenda U. (1995). The occurrence of C-H⋯O hydrogen bonds in proteins. J. Mol. Biol..

[107] Fabiola G.F., Krishnaswamy S., Nagarajan V., Pattabhi V. (1997). C-H⋯O hydrogen bonds in beta-sheets. Acta Crystallogr. Sect. D Struct. Biol..

[108] Bella J., Brodsky B., Berman H.M. (1995). Hydration Structure of a Collagen Peptide. Structure.

[109] Bella J., Berman H.M. (1996). Crystallographic evidence for C-alpha-H⋯O=C hydrogen bonds in a collagen triple helix. J. Mol. Biol..

[110] Kang B.S., Devedjiev Y., Derewenda U., Derewenda Z.S. (2004). The PDZ2 domain of syntenin at ultra-high resolution: Bridging the gap between macromolecular and small molecule crystallography. J. Mol. Biol..

[111] Laulumaa S., Kursula P. (2019). Sub-Atomic Resolution Crystal Structures Reveal Conserved Geometric Outliers at Functional Sites. Molecules.

[112] Addlagatta A., Krzywda S., Czapinska H., Otlewski J., Jaskolski M. (2001). Ultrahigh-resolution structure of a BPTI mutant. Acta Crystallogr. Sect. D Struct. Biol..

[113] Vargas R., Garza J., Dixon D.A., Hay B.P. (2000). How strong is the C-alpha-H⋯O=C hydrogen bond?. J. Am. Chem. Soc..

[114] Scheiner S., Kar T., Gu Y.L. (2001). Strength of the C^α^H··O hydrogen bond of amino acid residues. J. Biol. Chem..

[115] Scheiner S. (2006). Contributions of NH···O and CH···O hydrogen bonds to the stability of beta-sheets in proteins. J. Phys. Chem. B.

[116] Scheiner S. (2005). Relative strengths of NH··O and CH··O hydrogen bonds between polypeptide chain segments. J. Phys. Chem. B.

[117] Cordier F., Barfield M., Grzesiek S. (2003). Direct observation of C-alpha-H-alpha⋯O=C hydrogen bonds in proteins by interresidue (h3)J(CalphaC′) scalar couplings. J. Am. Chem. Soc..

[118] Manikandan K., Ramakumar S. (2004). The occurrence of C-H⋯O hydrogen bonds in alpha-helices and helix termini in globular proteins. Proteins-Struct. Funct. Bioinform..

[119] Mottamal M., Lazaridis T. (2005). The contribution of C-alpha-H⋯O hydrogen bonds to membrane protein stability depends on the position of the amide. Biochemistry.

[120] Senes A., Ubarretxena-Belandia I., Engelman D.M. (2001). The C alpha-H⋯O hydrogen bond: A determinant of stability and specificity in transmembrane helix interactions. Proc. Natl. Acad. Sci. USA.

[121] Senes A., Engel D.E., DeGrado W.F. (2004). Folding of helical membrane proteins: The role of polar, GxxxG-like and proline motifs. Curr. Opin. Struct. Biol..

[122] Dhar J., Chakrabarti P., Saini H., Raghava G.P.S., Kishore R. (2015). omega-Turn: A novel beta-turn mimic in globular proteins stabilized by main-chain to side-chain C-H⋯O interaction. Proteins-Struct. Funct. Bioinform..

[123] Otero L.H., Rojas-Altuve A., Llarrull L.I., Carrasco-Lopez C., Kumarasiri M., Lastochkin E., Fishovitz J., Dawley M., Hesek D., Lee M. (2013). How allosteric control of Staphylococcus aureus penicillin binding protein 2a enables methicillin resistance and physiological function. Proc. Natl. Acad. Sci. USA.

[124] Newberry R.W., Raines R.T. (2019). Secondary Forces in Protein Folding. ACS Chem. Biol..

[125] Buncel E., Clement O., Onyido I. (2000). Metal ion effects in isotopic hydrogen exchange in biologically important heterocycles. Acc. Chem. Res..

[126] Scheiner S., Kar T., Pattanayak J. (2002). Comparison of various types of hydrogen bonds involving aromatic amino acids. J. Am. Chem. Soc..

[127] Nanda V., Schmiedekamp A. (2008). Are aromatic carbon donor hydrogen bonds linear in proteins?. Proteins-Struct. Funct. Bioinform..

[128] Steinert R.M., Kasireddy C., Heikes M.E., Mitchell-Koch K.R. (2022). Newly identified C–H⋯O hydrogen bond in histidine. Phys. Chem. Chem. Phys..

[129] Derewenda Z.S., Derewenda U., Kobos P.M. (1994). (His)C epsilon-H⋯O=C < hydrogen bond in the active sites of serine hydrolases. J. Mol. Biol..

[130] Ash E.L., Sudmeier J.L., Day R.M., Vincent M., Torchilin E.V., Haddad K.C., Bradshaw E.M., Sanford D.G., Bachovchin W.W. (2000). Unusual H-1 NMR chemical shifts support (His) C-epsilon 1-H⋯O=C H-bond: Proposal for reaction-driven ring flip mechanism in serine protease catalysis. Proc. Natl. Acad. Sci. USA.

[131] Yasutake Y., Konishi K., Muramatsu S., Yoshida K., Aburatani S., Sakasegawa S.I., Tamura T. (2021). Bacterial triacylglycerol lipase is a potential cholesterol esterase: Identification of a key determinant for sterol-binding specificity. Int. J. Biol. Macromol..

[132] Petrella R.J., Karplus M. (2004). The role of carbon-donor hydrogen bonds in stabilizing tryptophan conformations. Proteins.

[133] Sekula B., Ruszkowski M., Dauter Z. (2020). S-adenosylmethionine synthases in plants: Structural characterization of type I and II isoenzymes from Arabidopsis thaliana and Medicago truncatula. Int. J. Biol. Macromol..

[134] Chakrabarti P., Chakrabarti S. (1998). C-H⋯O hydrogen bond involving proline residues in alpha-helices. J. Mol. Biol..

[135] Wishart D.S., Sykes B.D., Richards F.M. (1991). Relationship between Nuclear-Magnetic-Resonance Chemical-Shift and Protein Secondary Structure. J. Mol. Biol..

[136] Arnold M.R., Kremer W., Ludemann H.D., Kalbitzer H.R. (2002). H-1-NMR parameters of common amino acid residues measured in aqueous solutions of the linear tetrapeptides Gly-Gly-X-Ala at pressures between 0.1 and 200 MPa. Biophys. Chem..

[137] Bundi A., Wuthrich K. (1979). H-1-Nmr Parameters of the Common Amino-Acid Residues Measured in Aqueous-Solutions of the Linear Tetrapeptides H-Gly-Gly-X-L-Ala-Oh. Biopolymers.

[138] Guo H.B., Beahm R.F., Guo H. (2004). Stabilization and destabilization of the C-delta-h⋯O=C hydrogen bonds involving proline residues in helices. J. Phys. Chem. B.

[139] Vijay-Kumar S., Cook W.J. (1992). Structure of a sarcoplasmic calcium-binding protein from Nereis diversicolor refined at 2.0 A resolution. J. Mol. Biol..

[140] Daniecki N.J., Bhatt M.R., Yap G.P.A., Zondlo N.J. (2022). Proline C-H Bonds as Loci for Proline Assembly via C-H/O Interactions. ChemBioChem.

[141] Jiang L., Lai L. (2002). CH⋯O hydrogen bonds at protein-protein interfaces. J. Biol. Chem..

[142] Pierce A.C., Sandretto K.L., Bemis G.W. (2002). Kinase inhibitors and the case for CH⋯O hydrogen bonds in protein-ligand binding. Proteins.

[143] Panigrahi S.K. (2008). Strong and weak hydrogen bonds in protein-ligand complexes of kinases: A comparative study. Amino Acids.

[144] Ferguson F.M., Gray N.S. (2018). Kinase inhibitors: The road ahead. Nat. Rev. Drug Discov..

[145] Attwood M.M., Fabbro D., Sokolov A.V., Knapp S., Schioth H.B. (2021). Trends in kinase drug discovery: Targets, indications and inhibitor design. Nat. Rev. Drug Discov..

[146] Derewenda Z.S., Hawro I., Derewenda U. (2020). C–H⋯O hydrogen bonds in kinase-inhibitor interfaces. IUBMB Life.

[147] Xing L., Klug-Mcleod J., Rai B., Lunney E.A. (2015). Kinase hinge binding scaffolds and their hydrogen bond patterns. Bioorg. Med. Chem..

[148] Knowles P.P., Murray-Rust J., Kjaer S., Scott R.P., Hanrahan S., Santoro M., Ibanez C.F., McDonald N.Q. (2006). Structure and chemical inhibition of the RET tyrosine kinase domain. J. Biol. Chem..

[149] Bukrejewska M., Derewenda U., Radwanska M., Engel D.A., Derewenda Z.S. (2017). Crystal structures of the methyltransferase and helicase from the ZIKA 1947 MR766 Uganda strain. Acta Crystallogr. Sect. D Struct. Biol..

[150] Fanfrlik J., Kolar M., Kamlar M., Hurny D., Ruiz F.X., Cousido-Siah A., Mitschler A., Rezac J., Munusamy E., Lepsik M. (2013). Modulation of aldose reductase inhibition by halogen bond tuning. ACS Chem. Biol..

[151] Creeth J.M., Gulland J.M., Jordan D.O. (1947). Deoxypentose nucleic acids; viscosity and streaming birefringence of solutions of the sodium salt of the deoxypentose nucleic acid of calf thymus. J. Chem. Soc..

[152] Harding S.E., Channell G., Phillips-Jones M.K. (2018). The discovery of hydrogen bonds in DNA and a re-evaluation of the 1948 Creeth two-chain model for its structure. Biochem. Soc. Trans..

[153] Watson J.D., Crick F.H. (1953). Genetical implications of the structure of deoxyribonucleic acid. Nature.

[154] Wain-Hobson S. (2006). The third Bond. Nature.

[155] Pauling L., Corey R.B. (1956). Specific Hydrogen-Bond Formation between Pyrimidines and Purines in Deoxyribonucleic Acids. Arch. Biochem. Biophys..

[156] O’Brien E.J. (1963). The crystal structure of a complex of 9-Ethyl guanine with 1-Methyl cytosine. J. Mol. Biol..

[157] Hoogsteen K. (1959). The Structure of Crystals Containing a Hydrogen-Bonded Complex of 1-Methylthymine and 9-Methyladenine. Acta Crystallogr..

[158] Felsenfeld G., Rich A. (1957). Studies on the formation of two- and three-stranded polyribonucleotides. Biochim. Biophys. Acta.

[159] Courtois Y., Fromageot P., Guschlbauer W. (1968). Protonated polynucleotide structures. 3. An optical rotatory dispersion study of the protonation of DNA. Eur. J. Biochem..

[160] Quigley G.J., Ughetto G., van der Marel G.A., van Boom J.H., Wang A.H., Rich A. (1986). Non-Watson-Crick G.C and A.T base pairs in a DNA-antibiotic complex. Science.

[161] Leonard G.A., Mcauleyhecht K., Brown T., Hunter W.N. (1995). Do C-H⋯O Hydrogen-Bonds Contribute to the Stability of Nucleic-Acid Base-Pairs. Acta Crystallogr. Sect. D Struct. Biol..

[162] Starikov E.B., Steiner T. (1997). Computational support for the suggested contribution of C-H⋯O=C interactions to the stability of nucleic acid base pairs. Acta Crystallogr. Sect. D Struct. Biol..

[163] Asensio A., Kobko N., Dannenberg J.J. (2003). Cooperative hydrogen-bonding in adenine-thymine and guanine-cytosine base pairs. Density functional theory and Moller-Plesset molecular orbital study. J. Phys. Chem. A.

[164] Zhou P.P., Qiu W.Y. (2009). Red-shifted hydrogen bonds and blue-shifted van der Waals contact in the standard Watson-Crick adenine-thymine base pair. J. Phys. Chem. A.

[165] Parthasarathi R., Amutha R., Subramanian V., Nair B.U., Ramasami T. (2004). Bader’s and reactivity descriptors’ analysis of DNA base pairs. J. Phys. Chem. A.

[166] Yurenko Y.P., Zhurakivsky R.O., Samijlenko S.P., Hovorun D.M. (2011). Intramolecular CH⋯O hydrogen bonds in the AI and BI DNA-like conformers of canonical nucleosides and their Watson-Crick pairs. Quantum chemical and AIM analysis. J. Biomol. Struct. Dyn..

[167] Brovarets O.O., Yurenko Y.P., Hovorun D.M. (2014). Intermolecular CH⋯O/N H-bonds in the biologically important pairs of natural nucleobases: A thorough quantum-chemical study. J. Biomol. Struct. Dyn..

[168] Fick R.J., Liu A.Y., Nussbaumer F., Kreutz C., Rangadurai A., Xu Y., Sommer R.D., Shi H., Scheiner S., Stelling A.L. (2021). Probing the Hydrogen-Bonding Environment of Individual Bases in DNA Duplexes with Isotope-Edited Infrared Spectroscopy. J. Phys. Chem. B.

[169] Quintano M., Delgado A.A.A., Moura R.T., Freindorf M., Kraka E. (2022). Local mode analysis of characteristic vibrational coupling in nucleobases and Watson-Crick base pairs of DNA. Electron. Struct..

[170] Beiranvand N., Freindorf M., Kraka E. (2021). Hydrogen Bonding in Natural and Unnatural Base Pairs-A Local Vibrational Mode Study. Molecules.

[171] Hoshika S., Leal N.A., Kim M.J., Kim M.S., Karalkar N.B., Kim H.J., Bates A.M., Watkins N.E., SantaLucia H.A., Meyer A.J. (2019). Hachimoji DNA and RNA: A genetic system with eight building blocks. Science.

[172] Das S., Roy S., Bhattacharyya D. (2022). Understanding the role of non-Watson-Crick base pairs in DNA-protein recognition: Structural and energetic aspects using crystallographic database analysis and quantum chemical calculation. Biopolymers.

[173] Halder S., Bhattacharyya D. (2013). RNA structure and dynamics: A base pairing perspective. Prog. Biophys. Mol. Biol..

[174] Poltev V., Anisimov V.M., Dominguez V., Ruiz A., Deriabina A., Gonzalez E., Garcia D., Rivas F. (2021). Understanding the Origin of Structural Diversity of DNA Double Helix. Computation.

[175] Neidle S. (2021). Beyond the double helix: DNA structural diversity and the PDB. J. Biol. Chem..

[176] Bansal A., Kaushik S., Kukreti S. (2022). Non-canonical DNA structures: Diversity and disease association. Front. Genet..

[177] Schneider B., Neidle S., Berman H.M. (1997). Conformations of the sugar-phosphate backbone in helical DNA crystal structures. Biopolymers.

[178] Berman H.M. (1997). Crystal studies of B-DNA: The answers and the questions. Biopolymers.

[179] Yurenko Y.P., Zhurakivsky R.O., Samijlenko S.P., Ghomi M., Hovorun D.M. (2007). The whole of intramolecular H-bonding in the isolated DNA nucleoside thymidine. AIM electron density topological study. Chem. Phys. Lett..

[180] Berger I., Egli M., Rich A. (1996). Inter-strand C-H⋯O hydrogen bonds stabilizing four-stranded intercalated molecules: Stereoelectronic effects of 04′ in cytosine-rich DNA. Proc. Natl. Acad. Sci. USA.

[181] Auffinger P., LouiseMay S., Westhof E. (1996). Molecular dynamics simulations of the anticodon hairpin of tRNA(Asp): Structuring effects of C-H⋯O hydrogen bonds and of long-range hydration forces. J. Am. Chem. Soc..

[182] Brandl M., Lindauer K., Meyer M., Suhnel J. (1999). C-H⋯O and C-H⋯N interactions in RNA structures. Theor. Chem. Acc..

[183] Witts R.N., Hopson E.C., Koballa D.E., Van Boening T.A., Hopkins N.H., Patterson E.V., Nagan M.C. (2013). Backbone-Base Interactions Critical to Quantum Stabilization of Transfer RNA Anticodon Structure. J. Phys. Chem. B.

